# Relationship between body water distribution and sarcopenia in older adults: a systematic review

**DOI:** 10.1186/s11556-026-00402-4

**Published:** 2026-01-23

**Authors:** Weslley Barbosa Sales, Laura Beatriz Alves Costa, Melissa Silva Rocha Pereira, Sabrina Gabrielle Gomes Fernandes Mâcedo, Edgar Ramos Vieira, Álvaro Campos Cavalcanti Maciel

**Affiliations:** 1 Lagoa Nova, Natal - RN, Brazil, 59078-970, Federal University of Rio Grande do Norte, Natal, Brazil; 2 Florida International University, Miami, USA

**Keywords:** Bioelectrical impedance, Intracellular water, Extracellular water, Total body water, Sarcopenia

## Abstract

**Objective:**

To examine potential relationships between body water distribution, assessed by bioelectrical impedance analysis (BIA), and sarcopenia in older adults.

**Method:**

This systematic review was conducted based on the methodological guidelines established by the Preferred Reporting Items for Systematic Reviews and Meta-Analyses (PRISMA). Were Included observational studies exploring the relationship between intracellular water, extracellular water, and total body water – assessed by BIA and sarcopenia. The quality of the studies was assessed using the Joanna Briggs Institute (JBI) assessment tools and guidelines.

**Results:**

Initially, 16,056 articles were identified, and 11 studies were considered eligible for inclusion. Among the studies analyzed, five studies used the ECW/TBW ratio, examining its association with aspects related to sarcopenia, such as muscle strength, loss of muscle mass and physical performance in the older adults. The results indicated that the proportions of extracellular/total body water (ECW/TBW) and extracellular/intracellular water (ECW/ICW) are associated with sarcopenia, muscle strength, loss of muscle mass, and physical performance in the older people. Studies suggest that these relationships may be useful for understanding muscle health and the impact of aging. Most studies presented high methodological quality (above 70%), indicating a low risk of bias.

**Conclusions:**

The findings of this systematic review indicate that imbalances in body water distribution, particularly in the ECW/TBW and ECW/ICW ratios, are consistently associated with sarcopenia, loss of muscle mass, reduced strength, and poorer physical performance in the older adults.

## Introduction

The global aging process poses growing challenges to public health. Integrated strategies are increasingly needed to maintain functionality and quality of life in older adults [1]. Among these challenges, sarcopenia stands out. It is a geriatric syndrome marked by the progressive decline of muscle mass and strength, leading to reduced autonomy and greater vulnerability to falls, fractures, and disability [2].

Aging is also accompanied by notable changes in body composition. These include reduced lean mass, increased fat mass, and a decline in total body water [1]. Such alterations affect muscle health and physiological homeostasis [2]. Therefore, accurate diagnostic tools are essential for the early identification of risks associated with aging [2, 3].

BIA has become a practical, accessible, and non-invasive tool for assessing body composition [4]. Although lean mass, muscle mass index, and phase angle have been widely explored in relation to sarcopenia, and impedance ratio has increasingly been recognized as a direct indicator of cellular integrity and inflammatory status, fewer studies have examined body water compartments in detail [5, 6]. Research conducted between 2020 and 2022 has shown that ratios such as ECW/TBW can serve as prognostic markers in clinical populations, including individuals with cancer or frailty. However, evidence remains limited for community-dwelling older adults [5, 6]. A clearer understanding of ICW, ECW, and TBW in this population may provide valuable information about fluid balance, cellular integrity, and their potential relationships with muscle function [4–6].

However, evidence in older adults remains limited. Existing studies often have small samples, use heterogeneous methods, or lack standardized diagnostic criteria, including those proposed by the updated European Working Group on Sarcopenia in Older People (EWGSOP2) [5–7]. Beyond these methodological gaps, there remains uncertainty about how imbalances in body fluid distribution, such as changes in the extracellular-to-total body water or intracellular-to-extracellular water ratios, can be effectively applied in clinical practice. Understanding these patterns may help clinicians, physiotherapists, and nutritionists identify early signs of cellular dysfunction, fluid retention, or muscle deterioration, supporting more targeted interventions for the prevention and management of sarcopenia [2, 7, 8]. 

Given these gaps, this review aimed to examine potential relationships between body water distribution, assessed by bioelectrical impedance analysis, and sarcopenia in older adults. Changes in body fluid balance have been linked to inflammaging, a chronic low-grade inflammatory state associated with aging that may contribute to declines in muscle quality [8]. In this context, alterations in the balance between intracellular and extracellular water have been proposed as potential early markers of muscle frailty, reflecting compromised cellular integrity and reduced muscle function [7]. Understanding these hydration patterns may help identify non-invasive markers of risk and support more sensitive clinical strategies and personalized interventions aimed at healthy aging, particularly in primary care settings.

## Materials and methods

### Design of study

This systematic review was conducted based on the methodological guidelines established by the Preferred Reporting Items for Systematic Reviews and Meta-Analyses (PRISMA) [9–11]. The protocol of this study was previously registered on the PROSPERO (International Prospective Register of Systematic Reviews) platform [12], and assigned the CRD42023494500 approval code, ensuring traceability and commitment to the proposed methodology.

### Identification of the research question

The present systematic review was guided by the following central question: “Is there a relationship between body water components and sarcopenia in the older adults?”

To address this research question, the PECOT [11] framework was applied. The population comprised individuals aged 60 years or older. The exposure included studies that examined the relationship between sarcopenia and body water compartments, such as total body water, intracellular water, and extracellular water, assessed using bioelectrical impedance analysis. The comparison focused on differences in body water distribution between sarcopenic and non-sarcopenic older adults, based on measurements obtained using segmental multifrequency devices. The outcome of interest was the association between imbalances in body fluid composition and the presence of sarcopenia. Finally, the review included observational study designs, including cross-sectional, prospective, and retrospective studies.

#### Identification of relevant studies

This review included observational studies, such as cross-sectional, prospective (longitudinal), and retrospective designs, with no restrictions on language or year of publication. Eligible studies focused on the relationship between body water compartments (intracellular, extracellular, and total), measured by BIA, and sarcopenia in older adults. Studies were excluded if they focused on populations with conditions that could confound the association between water distribution and sarcopenia, such as cancer, cardiovascular disease, respiratory disorders, hematologic conditions, hepatic dysfunction, or neurological diseases.

A comprehensive search was conducted in the following electronic databases: PubMed, Scopus, Web of Science, Embase, BVS, Portal CAPES, ScienceDirect and SciELO. The search strategy combined controlled vocabulary and free-text terms, using the following descriptors: “intracellular water” OR “extracellular water” OR “total body water” AND “sarcopenia”. Further details regarding the specific search strategies applied to each database are available in Table 1.

**Table 1:** Full search strategies used in the electronic databases

Databases	Search strategy
PubMed (MEDLINE)	(“Body Water“[MeSH] OR “Intracellular Fluid“[MeSH] OR “Extracellular Fluid“[MeSH] OR “intracellular water” OR “extracellular water” OR “total body water”) AND (“Sarcopenia“[MeSH] OR sarcopenia)
Embase	(‘body water’/exp OR ‘intracellular fluid’/exp OR ‘extracellular fluid’/exp OR ‘intracellular water’ OR ‘extracellular water’ OR ‘total body water’) AND (‘sarcopenia’/exp OR sarcopenia)
Scopus	TITLE-ABS-KEY (“intracellular water” OR “extracellular water” OR “total body water”) AND TITLE-ABS-KEY (sarcopenia)
Web of Science	TS=(“intracellular water” OR “extracellular water” OR “total body water”) AND TS=(sarcopenia)
ScienceDirect	(“intracellular water” OR “extracellular water” OR “total body water”) AND (sarcopenia)
BVS (MEDLINE/LILACS)	((“água corporal total” OR “água intracelular” OR “água extracelular”) OR (“total body water” OR “intracellular water” OR “extracellular water”)) AND (sarcopenia)
SciELO	(“intracellular water” OR “extracellular water” OR “total body water”) AND (sarcopenia)
Portal CAPES	(“intracellular water” OR “extracellular water” OR “total body water”) AND (sarcopenia)

Additionally, the reference lists of all included studies and relevant gray literature (theses, dissertations, and conference proceedings) were screened to identify additional eligible studies. This step was carried out by an additional researcher to ensure thoroughness and reduce selection bias.

Experimental studies, reviews (systematic or narrative), editorials, protocols, theoretical essays, and articles without abstract or full-text availability were excluded.

### Study selection

The study selection process was conducted in two stages using the Rayyan platform (https://rayyan.qcri.org.), which facilitates the collaborative and systematic screening of publications (https://rayyan.qcri.org) [13]. Rayyan (Qatar Computing Research Institute) was used solely to support manual screening and reference management. No artificial intelligence–based tools or automated functionalities were employed during screening, study selection, or data extraction. Initially, the titles and abstracts of the eligible articles were reviewed and, in the second stage, the full texts of the selected studies were read.

Both stages were performed independently by two reviewers previously trained in the eligibility criteria. In cases of disagreement or doubt, a third reviewer was called to ensure consensus and consistency of decisions.

### Data extraction

Data extraction was performed using a custom spreadsheet to collect key information, including authors, year of publication, country, study objective, design, assessment method for sarcopenia, and key outcomes. This tool was tested by reviewers to ensure accuracy. Data extraction was conducted in pairs, with two reviewers independently extracting data from all included studies. Any disagreements were resolved through discussion, and when consensus was not reached, a third reviewer was consulted.

### Methodological quality

The quality of the studies was assessed using the Joanna Briggs Institute (JBI) assessment tools and guidelines [14]. This evaluation aimed to assess methodological rigor and identify possible biases in the design, conduction, and analysis of the study.

The studies were classified according to the proportion of positive responses to the items assessed: high risk of bias (≤ 49%), moderate risk (50%–69%), and low risk of bias (≥ 70%). The evaluation was carried out independently by two reviewers, and the divergences were resolved by a third member of the team, ensuring the impartiality and robustness of the judging process.

## Results

**Fig. 1:**
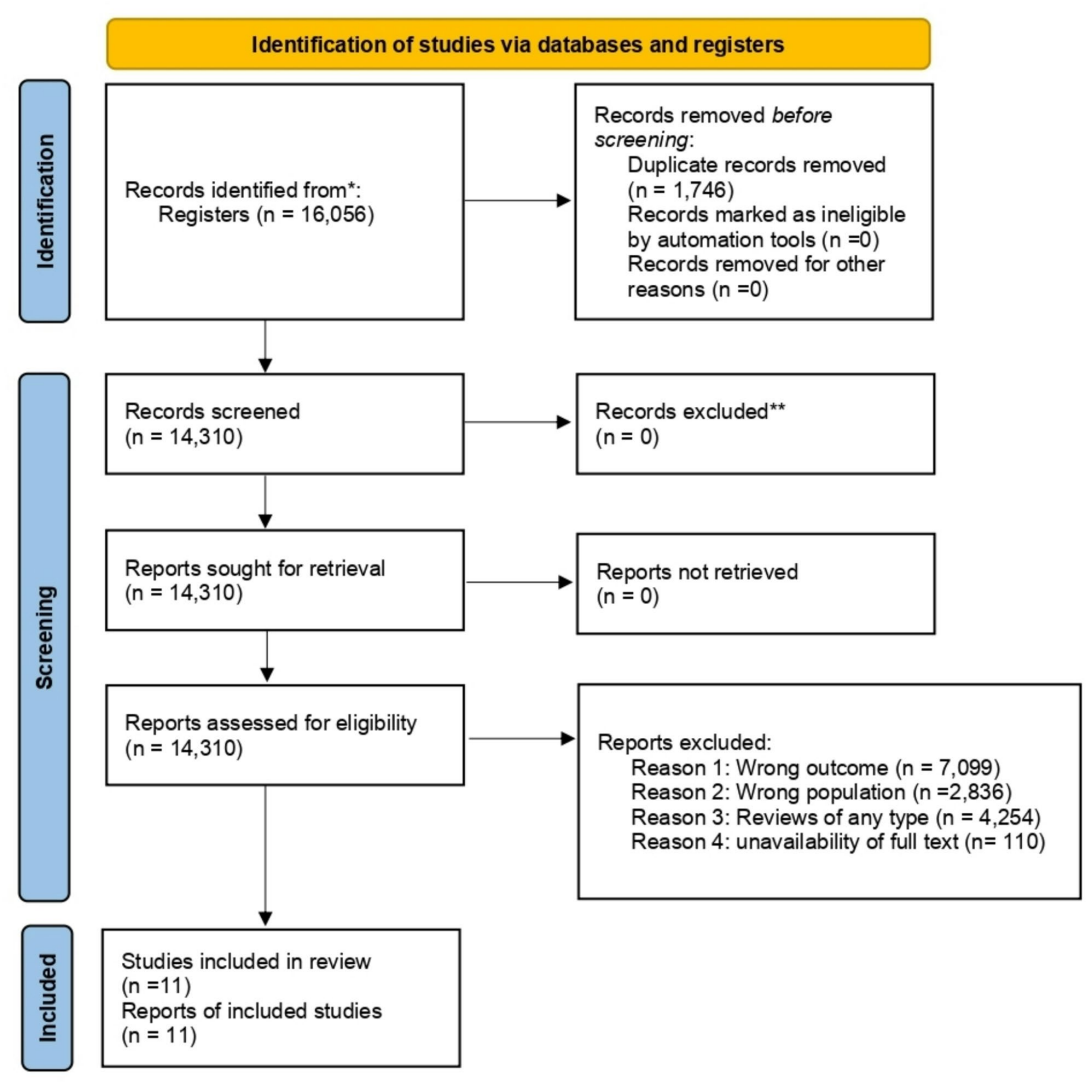
PRISMA flowcharts: methodological path of the systematic study selection review

All database searches and record retrieval were conducted on August 12, 2025. All databases were searched on the same day to ensure consistency and to prevent discrepancies related to studies published between search dates. A total of 16,056 articles were identified from the platforms used and from the search in the gray literature. After the removal of duplicates, 1,746 articles were excluded, resulting in 14,310 articles whose titles and abstracts were reviewed. Of these, 11 articles were considered eligible for this review. The stages of the selection process are illustrated in the PRISMA flowchart (Fig. 1), while the main characteristics of the included studies are presented in Table 2, and the methodological quality and risk of bias assessments are summarized in Table 3.

**Table 2:** Characterization of cross-sectional studies on body water in sarcopenia assessment (*N* = 11)

Authors (year)	Country	Analyzed water variable	Objective	Evaluation method (Sarcopenia)	Main results
Yamada et al. (2010) [15]	Japan	Extracellular water	Investigate the relationship between intracellular and extracellular water volume and lower limb muscle strength, and the influence of aging on this relationship.	Handgrip strength (dynamometer), muscle mass (BIA), physical performance (gait speed or SPPB).	With aging, the extracellular water-to-total body water ratio increases, potentially masking muscle loss. Assessing the intracellular water proportion improves the link between muscle mass and strength, making the extracellular water/total body water ratio crucial for evaluating muscle strength in older adults.
Yamada et al. (2016) [16]	Japan	Extracellular and Intracellular Water Ratio	Investigate the relationship between ECW/ICW in the upper legs and knee extension strength and gait speed in community-dwelling older adults, as well its potential as a biomarker for muscle.	Handgrip strength (dynamometer), muscle mass (BIA), physical performance (gait speed or SPPB).	The ECW/ICW ratio in the thighs is negatively correlated with knee extension strength and gait speed, indicating that a higher ratio reflects reduced muscle quality, making it a potential biomarker for age-related muscle decline.
Taniguchi et al. (2017) [17]	Japan	Extracellular-to-intracellular water ratio	The study aims to investigate whether the echo index (EI) and the ECW/ICW independently influence muscle strength in older women.	Handgrip strength (dynamometer), muscle mass (BIA), physical performance (gait speed or SPPB).	The results indicate that muscle strength in older women is positively correlated with muscle thickness (MT) and negatively correlated with both the echo index (EI) and ECW/ICW. These findings highlight the potential of these parameters as valuable tools for assessing muscle quality, particularly in the context of age-related muscle weakness.
Serra-Prat et al. (2019) [18]	Spain	Intracellular water ratio	Evaluate the relationship between intracellular water (ICW) content in lean mass and muscle strength, functional capacity, frailty, and other clinical characteristics in older people	Handgrip strength (dynamometer), muscle mass (BIA), physical performance (gait speed or SPPB).	The findings reveal that in older adults, higher intracellular water content in lean mass is associated with greater muscle strength, improved functional capacity, and a reduced risk of frailty. This positive relationship remains significant even after adjusting for age, sex, and health conditions, underscoring the importance of intracellular water for maintaining muscle and functional health in aging populations.
Tanaka et al. (2020) [19]	Japan	Extracellular-tototal body water ratio	Investigate the relationships between the extracellular-tototal body water ratio (ECW/TBW) and locomotive syndrome, frailty, and sarcopenia in older people.	Handgrip strength (dynamometer), muscle mass (BIA), physical performance (gait speed or SPPB).	An increase in the ECW/TBW ratio may reflect the risk of locomotive syndrome and frailty but does not appear to be related to sarcopenia, making this ratio an important indicator in bioelectrical impedance analysis for assessing these conditions.
Park et al. (2021) [20]	South Korea	Extracellular water-to-total body water	Evaluate the association between sarcopenia and the extracellular-tototal body water ratio (ECW/TBW), one of the outcomes of bioimpedance that can be easily measured in community settings.	Handgrip strength (dynamometer), muscle mass (BIA), physical performance (gait speed or SPPB).	The study suggests that the ECW/TBW ratio may be a valid indicator for assessing physical function in older people, according to the recently revised sarcopenia criteria.
Hioka et al. (2021) [21]	Japan	Extracellular-to-intracellular water ratio	Investigate the relationship between handgrip strength and gait speed with the ECW/ICW in communitydwelling older women.	Handgrip strength (dynamometer), muscle mass (BIA), physical performance (gait speed or SPPB).	The findings showed that a higher ECW/ICW in older women was linked to reduced handgrip strength and slower gait speed. Furthermore, the total ECW/ICW ratio remained significantly and independently associated with handgrip strength after adjusting for confounding factors.
Hioka et al. (2022) [22]	Japan	Extracellular water-to-total body water	Assess the relationships between the extracellular-tototal body water ratio (ECW/TBW) and handgrip strength, gait speed, and skeletal muscle index (SMI) in older women, in order to understand how ECW/TBW may impact the assessment of sarcopenia.	Handgrip strength (dynamometer), muscle mass (BIA), physical performance (gait speed or SPPB).	The results revealed that the skeletal muscle index (SMI) was associated with handgrip strength but not with gait speed in older adults. In participants with an extracellular-to-total body water (ECW/TBW) ratio below 0.40, SMI correlated with handgrip strength but not with gait speed. However, in those with an ECW/TBW ratio of 0.40 or higher, SMI was not linked to either variable.
Hirata et al. (2022) [23]	Japan	Intracellular water to total body water ratio	The study investigated the relationship between age-related differences in the intracellular water to total body water ratio (ICW/TBW) and the explosive strength of the plantar flexors.	Handgrip strength (dynamometer), muscle mass (BIA), physical performance (gait speed or SPPB).	With aging, the intracellular water to total body water ratio (ICW/TBW) decreases, which correlates with reduced rapid muscle strength development (RTD) and increased time to peak force (TPT). ICW/TBW, as assessed via electromyography (EMG), is positively associated with RTD and negatively with TPT, though it shows no correlation with muscle activity. These findings suggest that reduced ICW/TBW reflects age-related changes in muscle contractile properties.
Iwasaka et al. (2023) [7]	Japan	Extracellular-to intracellular water ratio	Investigate agerelated changes, gender differences, the contribution to muscle strength independent of skeletal muscle mass (SMM), and potential improvements through physical activity in ECW/ICW as a marker of muscle quality.	Handgrip strength (dynamometer), muscle mass (BIA), physical performance (gait speed or SPPB).	The results indicated that the ECW/ICW ratio increases with age, particularly in men. This ratio is associated with handgrip strength and is influenced by physical activity levels. This suggests that the ECW/ICW ratio may be an indicator of muscle quality and that regular physical exercise could improve it.
Hioka et al. (2025) [24]	Japan	Extracellular water-to-total body water ratio	This study aimed to clarify the relationship between the SARC-F score and the extracellular water-to-total body water ratio (ECW/TBW) in community-dwelling older people.	Handgrip strength (dynamometer), muscle mass (BIA), physical performance (gait speed or SPPB) and used the SARC-F as a screening test for sarcopenia diagnosis.	This study revealed a positive correlation between SARC-F score and ECW/TBW in community-dwelling older people, and this relationship persisted even after adjusting for confounding factors.

*BIA* Bioelectrical Impedance Analysis, *ICW* Intracellular Water, *ECW* Extracellular Water, *TBW* Total Body Water, *ECW/ICW* Extracellular Water to Intracellular Water Ratio, *ECW/TBW* Extracellular Water to Total Body Water Ratio, *SPPB* Short Physical Performance Battery, *SMI* Skeletal Muscle Index, *RTD* Rate of Torque Development, *TPT* Time to Peak Torque

**Table 3:** Technical characteristics of the included cross-sectional studies, including sample descriptors, BIA devices, measurement specifications, prediction equations, and outcomes assessed (*N* = 11)

Authors and year	Sample (*n*; sex; age)	BIA device	Frequency/Type	Prediction equations used	Outcomes reported
Yamada et al. (2010) [15]	*n* = 178 (M/F), 72.3 ± 5.4 y	InBody 720	Multifrequency (1–1000 kHz)	Manufacturer equation (InBody proprietary)	ICW, ECW, TBW, ECW/ICW
Yamada et al. (2016) [16]	*n* = 312 (M/F), 73.1 ± 6.2 y	InBody S10	Multifrequency (1–1000 kHz)	InBody proprietary equation	ICW, ECW, TBW, ECW/TBW
Taniguchi et al. (2017) [17]	*n* = 142 (F), 75.4 ± 6.8 y	Tanita MC-780	Multifrequency (5–250 kHz)	Tanita predictive equations	ICW, ECW, TBW
Serra-Prat et al. (2019) [18]	*n* = 258 (M/F), 84.2 ± 5.1 y	ImpediMed SFB7	Bioimpedance spectroscopy	Cole–Cole modelling	ICW/ECW ratio
Tanaka et al. (2020) [19]	*n* = 196 (M/F), 70.8 ± 4.9 y	InBody 770	Multifrequency (1–1000 kHz)	Manufacturer equation	ICW, ECW, TBW
Park et al. (2021) [20]	*n* = 430 (M/F), 71.6 ± 6.1 y	Tanita MC-980	Multifrequency	Tanita equation	ICW, ECW, TBW, ECW/TBW
Hioka et al. (2021) [21]	*n* = 128 (F), 80.1 ± 7.0 y	InBody S10	Multifrequency	InBody proprietary	ECW, ICW, ECW/TBW
Hioka et al. (2022) [22]	*n* = 165 (M/F), 81.5 ± 6.3 y	InBody S10	Multifrequency	InBody proprietary	ECW, ICW, TBW
Hirata et al. (2022) [23]	*n* = 90 (M/F), 74.9 ± 5.7 y	ImpediMed SFB7	BIS	Cole–Cole modelling	ICW, ECW, ECW/ICW
Iwasaka et al. (2023) [7]	*n* = 220 (M/F), 76.3 ± 6.5 y	Tanita MC-780	Multifrequency	Tanita equations	ICW, ECW, TBW
Hioka et al. (2025) [24]	*n* = 109 (F), 82.4 ± 6.9 y	InBody 770	Multifrequency	InBody proprietary	ICW, ECW, ECW/TBW, ECW/ICW

Bioelectrical Impedance Analysis; *MF-BIA* Multifrequency BIA, *BIS* Bioimpedance Spectroscopy, *ICW* Intracellular Water, *ECW* Extracellular Water, *TBW* Total Body Water, *M* Men, *F* Women, *M/F* Mixed Sample (Men and Women), *SD* Standard Deviation

### Sample characterization

The systematic review included 11 observational studies that investigated the relationship between body water compartments and sarcopenia in older adults. Most studies were conducted in Japan (*n* = 9), with a study conducted in South Korea (*n* = 1) and another in Spain (*n* = 1). The samples varied widely in size, ranging from 50 to 8.018 participants, with a predominance of community-dwelling older adults.

Most studies have focused on the analysis of the ECW/ICW and the ECW/TBW as potential markers of muscle quality, strength, and sarcopenia.

Methods of assessing sarcopenia varied across studies. Most studies evaluated muscle strength using handgrip dynamometry, muscle mass through BIA, and physical performance using tests such as the gait speed test or Short Physical Performance Battery (SPPB). Diagnostic criteria were based on guidelines from the EWGSOP (2010) and EWGSOP2 (2019) and the Asian Working Group for Sarcopenia (2019). One study used the SARC-F [24] questionnaire as a screening tool [1]. 

In general, the findings demonstrated that an increase in the ECW/ICW and/or ECW/TBW ratio were associated with reduced muscle strength and decreased gait speed [16, 21]. In addition, studies have highlighted the importance of intracellular water (ICW) in maintaining muscle function and strength, with lower ICW/TBW ratios correlating with declines in rapid muscle strength development and increased time to peak strength in older subjects [15, 16, 18]. 

### Water variables analyzed

The body water distribution variables investigated in the included studies showed methodological diversity. Five studies analyzed the ratio between extracellular and total water (ECW/TBW), focusing on its association with indicators of sarcopenia, such as loss of muscle mass, reduced strength, and impairment of physical performance [19–24]. 

Four studies investigated the ratio of ECW/ICW, exploring its association with muscle strength and muscle quality [7, 16, 18, 21]. The proportion of extracellular water was addressed separately in one study, focusing on its relationship with intracellular water content in lean mass, muscle strength, and gait speed in the older people. Another study specifically looked at intracellular water (ICW), examining its relationship with ECW volumes and lower limb strength, as well as the effects of aging on this dynamic.

### Methodological quality and risk of bias

The methodological quality and risk of bias of the included studies were assessed using eight predefined criteria. These criteria evaluated aspects such as study design, reliability of the measurement, control of confounding factors, and statistical analysis.

All studies achieved a quality methodological score above 70%, indicating low risk of bias. These studies demonstrated consistent methodological rigor, particularly in exposure measurement, outcome evaluation, and statistical analysis. However, some studies scored slightly lower (75%), mainly due to limitations in identifying confounding factors and control strategies.

In general, the included studies presented satisfactory methodological quality, ensuring the reliability of the findings of this review. This assessment helps clarify the evidence on how imbalances in body fluid distribution, particularly the extracellular-to-intracellular water and extracellular-to-total body water ratios, relate to muscle outcomes in older adults, including muscle strength, physical function, and the risk of sarcopenia. Detailed results of this analysis are presented in Table 4.

**Table 4:** Degree of methodological quality and risk of bias (*N* = 11)

Authors and year	#1	#2	#3	#4	#5	#6	#7	#8	Total	Methodological quality (%)	Risk of bias
Yamada et al. (2010) [15]	1	1	1	0	1	1	1	1	7	87.5%	Low
Yamada et al. (2016) [16]	1	1	1	0	1	1	1	1	7	87.5%	Low
Taniguchi et al. (2017) [17]	1	1	1	0	1	1	0	1	6	75.0%	Low
Serra-Prat et al. (2019) [18]	1	1	1	0	1	1	0	1	6	75.0%	Low
Tanaka et al. (2020) [19]	1	1	1	0	1	1	1	1	7	87.5%	Low
Park et al. (2021) [20]	1	1	1	0	1	1	1	1	7	87.5%	Low
Hioka et al. (2021) [21]	1	1	1	0	1	1	1	0	6	75.0%	Low
Hioka et al. (2022) [22]	1	1	1	0	1	1	1	0	6	75.0%	Low
Hirata et al. (2022) [23]	1	1	1	0	1	1	1	1	7	87.5%	Low
Iwasaka et al. (2023) [7]	1	1	1	0	1	1	1	1	7	87.5%	Low
Hioka et al. (2025) [24]	1	1	1	0	1	1	1	1	7	87.5%	Low

The risk of bias assessment was conducted using the appropriate JBI Critical Appraisal Checklist, which consists of eight items rated as Yes, No, Unclear, or Not applicable. The evaluated criteria were: #1 whether the criteria for inclusion in the sample were clearly defined; #2 whether the study subjects and the setting were described in detail; #3 whether the exposure was measured in a valid and reliable way; #4 whether objective and standard criteria were used for measurement of the condition; #5 whether confounding factors were identified; #6 whether strategies to deal with confounding factors were stated; #7 whether the outcomes were measured in a valid and reliable way; and #8 whether appropriate statistical analysis was used

0 No, 1 Yes, *NA* Not applicable

## Discussion

The findings of this systematic review provide consistent evidence supporting the association between alterations in body water distribution and sarcopenia in the older adults, emphasizing the influence of cellular hydration status on the maintenance of muscle integrity and functionality in the aging process. The findings reinforce the hypothesis that a reduction in ICW, accompanied by an increase in ECW, is one of the key features in the pathophysiological process that leads to sarcopenia. Specifically, the ECW/ICW ratio has been associated with muscle quality [7, 16] and strength [21, 25], while the ECW/TBW ratio has been associated with muscle mass loss [19, 20] and decline in physical performance [22–24].

Our results align with previous studies demonstrating that lower levels of ICW are strongly associated with reduced muscle mass and strength [24, 26, 27]. Given that the ICW constitutes a substantial portion of muscle mass, its decline signals muscle atrophy and impairment of cellular homeostasis [18, 28]. Increasing the proportion of ECW, on the other hand, suggests fluid redistribution, which may be linked to inflammatory processes, decreased capillary permeability, and altered sodium-potassium balance, all of which are implicated in muscle deterioration [29, 30]. 

From a pathophysiological perspective, several mechanisms associated with aging may underlie the relationship between water compartment imbalances and sarcopenia. One of the most critical changes is the progressive decline in total body water, particularly ICW, which is closely linked to cellular volume, membrane integrity, and metabolic activity. Muscle tissue, comprising predominantly ICW, is especially vulnerable to such alterations, and a decrease in ICW reflects not only muscle mass reduction but also a loss in cellular function and hydration status [18, 28, 31]. 

Likewise, age-related cellular senescence and mitochondrial dysfunction contribute to reduced protein synthesis, impaired muscle regeneration, and increased oxidative stress, all of which lead to sarcopenic deterioration [1, 3]. These cellular alterations may be exacerbated by changes in the ionic environment and transmembrane gradients, particularly involving sodium and potassium fluxes, which are crucial for maintaining osmotic equilibrium and cellular excitability. A shift toward greater ECW content may reflect compromised capillary permeability, endothelial dysfunction, and increased systemic inflammation—phenomena widely observed in aging and chronic disease states [2, 4]. 

Furthermore, the chronic low-grade inflammation often seen in older adults (inflammaging) is associated with alterations in vascular and interstitial compartments, favoring fluid retention in the extracellular space [3, 15, 16]. This proinflammatory milieu, characterized by elevated levels of cytokines such as IL-6 and TNF-α, disrupts muscle protein turnover and promotes catabolic pathways, thereby compounding muscle wasting [1, 2, 31]. Increased ECW is also thought to interfere with nutrient diffusion and metabolite clearance, further impairing muscle cell metabolism and repair capacity [2, 31]. 

Additionally, the reduced ICW/TBW ratio seen in older individuals may reflect changes in hormonal signaling, including declines in anabolic hormones such as growth hormone, insulin-like growth factor-1 (IGF-1), and testosterone, which play central roles in muscle anabolism and water retention within cells [3, 4]. These endocrine alterations may decrease the osmotic drive necessary to maintain cellular hydration and protein synthesis in muscle fibers, thereby contributing to sarcopenia [15]. 

From a clinical perspective, these findings highlight the value of including body water compartment analysis, particularly the balance between intracellular and extracellular water, as part of a comprehensive assessment of sarcopenia. BIA provides a practical, non-invasive, and cost-effective means of evaluating these variables, offering additional insights beyond traditional measures of muscle mass and function [7, 29, 30]. Specifically, the ECW/TBW ratio has been recognized as a reliable surrogate marker of both cellular hydration and systemic inflammatory status, which are increasingly viewed as pivotal elements in the development and progression of sarcopenia [31, 32]. 

Previous studies indicate that body water variables obtained by bioelectrical impedance analysis, including total body water, intracellular water, and extracellular water, often show collinearity. This overlap can make it challenging to interpret the isolated contribution of each compartment to muscle strength and physical function. For this reason, many studies have shifted toward analyzing combined indicators, particularly ratios that offer a clearer picture of water distribution. Among these, the ECW/TBW ratio has gained prominence as a clinically relevant marker [19, 20, 22, 24]. It reflects inflammation, cellular hydration and overall metabolic status, aspects that are closely linked to muscle quality in older adults [8]. Because of this, it has been increasingly recommended as an auxiliary parameter in sarcopenia assessment and in rehabilitation contexts [8, 29]. 

In summary, the age-related shift from intracellular to extracellular water may reflect broader physiological and structural changes, such as inflammation, cellular dysfunction, and reductions in muscle quality, which may negatively affect physical performance [33, 34]. Monitoring these alterations through body water analysis may contribute to the early detection of sarcopenia and support interventions aimed at preserving muscle health [35]. Strengthening the monitoring of body water distribution using body composition analysis may help reduce the risk of frailty and disability and support functional independence in older adults [35, 36].

### Implications, limitations and future prospects

Despite the systematic approach of this review, some limitations should be acknowledged. The main limitation relates to the variability in the diagnostic criteria for sarcopenia across the included studies. Different definitions, such as those proposed by the EWGSOP (2019), the Asian Working Group for Sarcopenia (2019), and the SARC-F questionnaire, rely on distinct cutoff points and differ in their sensitivity, specificity, and practical focus [1]. These differences may lead to the identification of non-equivalent sarcopenic phenotypes, thereby affecting the comparability of outcomes and potentially introducing bias into the synthesized associations. Moreover, no subgroup analysis based on diagnostic criteria was performed, which limits our ability to isolate the effects of definitional variability. We recommend that future studies and reviews explore stratified analyses or meta-regression techniques to better account for this source of heterogeneity.

Another important limitation relates to the geographic concentration of the studies included. A large proportion of the data synthesized originated from Japan (9 out of 11 studies), which may limit the generalizability of our findings to other populations. Given that body water distribution patterns can vary significantly across ethnic groups due to genetic, cultural, and environmental factors, the applicability of ECW/ICW and ECW/TBW ratios as universal biomarkers for sarcopenia must be interpreted with caution. We suggest that future research prioritize greater ethnic and regional diversity in study samples to improve the external validity and global relevance of these biomarkers.

In addition, the variation in protocols used to measure body composition via BIA may have influenced the comparability and consistency of the findings. While some studies reported fluid distribution ratios such as ICW/ECW or ECW/TBW, others focused on individual compartments such as ICW or ECW. This methodological diversity, including differences in BIA devices, frequency settings, and predictive equations, adds further complexity to cross-study comparisons and interpretation.

Furthermore, the potential collinearity between isolated water compartments (ECW, ICW, and TBW) and their derived ratios (e.g., ECW/ICW and ECW/TBW) may have influenced the interpretation of individual associations, especially in multivariate models [15, 16, 18, 19, 29], This collinearity is expected, given the mathematical dependency between these variables, and may obscure unique contributions to sarcopenia-related outcomes [29]. Nevertheless, the emergence of ratio-based indicators can offer added value by capturing functional imbalances not evident in isolated measures. To better understand these complex relationships, future research could benefit from statistical techniques such as path analysis, which allows for the modeling of direct and indirect effects among interrelated variables. Such approaches may clarify the role of fluid distribution in sarcopenia and help improve the interpretability and robustness of findings in this field.

Overall, all included studies achieved methodological quality scores above 70%, indicating a generally low risk of bias, particularly in domains related to exposure measurement, outcome assessment, and statistical analysis. Minor reductions in scores were observed in some studies, mainly due to limitations in the identification and control of confounding factors. In addition, when full-text articles were not available, the authors were not contacted to request additional information, which can be considered a limitation of this review.

Another relevant aspect is the predominance of cross-sectional designs among the studies analyzed, which limit the causal inference between fluid imbalances and muscle outcomes.

Given these limitations, future research should address these gaps through longitudinal studies with diverse populations to clarify the dynamic interactions between hydration status and muscle aging. In addition, it is recommended to use statistical approaches that minimize collinearity effects in order to ensure more robust and comparable analyses, especially in investigations on the components of body water, such as intracellular, extracellular and total water.

### Final considerations

This review highlights that age-related changes in body fluid composition are directly implicated in the loss of muscle mass and function. The analysis of proportions such as ECW/ICW and ECW/TBW, as well as the isolated monitoring of ECW and ICW, emerges as a promising strategy to improve the clinical assessment of muscle health in the older people.

These hydroelectrolytic biomarkers, accessible through bioelectrical impedance, complement traditional methods of assessing, tracking, and diagnosing sarcopenia. However, the absence of standardized measurement protocols, validated cut-off points, and guidance on integrating these ratios into routine clinical workflows represents a critical gap. Given the variability in BIA equipment, measurement conditions (e.g., hydration status, posture), and analytical procedures across studies, future research should prioritize methodological standardization to ensure reproducibility and clinical applicability. Only through such standardization can these biomarkers evolve from promising research indicators into reliable, clinically actionable tools. 

Their incorporation into clinical practice—once standardized—can favor the development of more personalized prevention and management strategies, contributing to the preservation of functionality, the promotion of healthy aging, and the improvement of the quality of life of the older population.

## Data Availability

No datasets were generated or analysed during the current study.
